# Damage Localization and Severity Assessment of a Cable-Stayed Bridge Using a Message Passing Neural Network

**DOI:** 10.3390/s21093118

**Published:** 2021-04-30

**Authors:** Hyesook Son, Van-Thanh Pham, Yun Jang, Seung-Eock Kim

**Affiliations:** 1 Computer Engineering and Convergence Engineering for Intelligent Drone, Sejong University, Seoul 05006, Korea; atieer@naver.com; 2 Civil and Environmental Engineering, Sejong University, Seoul 05006, Korea; phamthanhwru@gmail.com (V.-T.P.); sekim@sejong.ac.kr (S.-E.K.)

**Keywords:** SHM, graph, MPNN, deep learning

## Abstract

Cable-stayed bridges are damaged by multiple factors such as natural disasters, weather, and vehicle load. In particular, if the stayed cable, which is an essential and weak component of the cable-stayed bridge, is damaged, it may adversely affect the adjacent cables and worsen the bridge structure condition. Therefore, we must accurately determine the condition of the cable with a technology-based evaluation strategy. In this paper, we propose a deep learning model that allows us to locate the damaged cable and estimate its cross-sectional area. To obtain the data required for the deep learning training, we use the tension data of the reduced area cable, which are simulated in the Practical Advanced Analysis Program (PAAP), a robust structural analysis program. We represent the sensor data of the damaged cable-stayed bridge as a graph composed of vertices and edges using tension and spatial information of the sensors. We apply the sensor geometry by mapping the tension data to the graph vertices and the connection relationship between sensors to the graph edges. We employ a Graph Neural Network (GNN) to use the graph representation of the sensor data directly. GNN, which has been actively studied recently, can treat graph-structured data with the most advanced performance. We train the GNN framework, the Message Passing Neural Network (MPNN), to perform two tasks to identify damaged cables and estimate the cable areas. We adopt a multi-task learning method for more efficient optimization. We show that the proposed technique achieves high performance with the cable-stayed bridge data generated from PAAP.

## 1. Introduction

Cable-stayed bridges, one of the essential transportation infrastructures in modern society, are damaged and corroded by external environments such as natural disasters, climate, ambient vibrations, and vehicle loads. As damage accumulates, the condition of the structure deteriorates, and the bridge loses its function. Damaged bridges even lead to collapse, causing severe problems such as human injury and economic loss. In particular, the stayed cable is a necessary but vulnerable primary component of cable-stayed bridges [1]. When the cable starts to be damaged, the stiffness and cross-sectional area decrease [2]. Since the cable has a small cross-sectional area, it may be lost due to low resistance against accidental lateral loads. The cable loss may cause overloading in the bridge and adversely affect adjacent cables [3]. Therefore, we must thoroughly inspect the cable conditions. However, we cannot directly know the damaged cable and its cross-sectional area only with raw data collected from the sensors on the bridge, such as the cable tension. Furthermore, if the damage degree is not significant, it may be challenging to determine whether the damage occurs visually, unlike cracks detection. Manual checking of all cables one by one is very inefficient and increases maintenance costs. Therefore, to ensure the safety and durability of the bridge, we need a technology-based evaluation strategy. Moreover, the technology must be able to capture small changes in the cable area accurately.

The importance of Structural Health Monitoring (SHM) has been emphasized to assess damage such as corrosion, defects, cracks, and material changes in structures. Researchers have introduced deep learning models as well as statistical analysis and machine learning as SHM techniques to determine the damaged cable locations [2] or detect stiffness reduction [4]. With the advancement of the device fabrication process, artificial intelligence meets the need for fast and accurate problem solving using vast amounts of data collected from sensor devices [5]. Deep learning models learn high-level representations of data and complex nonlinear correlations, which are frequently preferred as an automatic damage pattern prediction tool. In many civil engineering studies, deep learning models have achieved high performance with data-driven SHM techniques. Deep learning contributes to the advancement of SHM analysis because it effectively processes both unstructured data such as images and structured data such as time-series data. As SHM technologies, many researchers have proposed architectures such as Convolutional Neural Network (CNN), Recurrent Neural Network (RNN), Deep Autoencoder (DAE), and generative adversarial network (GNN) [6]. Pathiragea et al. [7] trained the autoencoder neural network to perform dimensionality reduction and estimated the stiffness element of the steel frame structure with modal information. Gu et al. [8] calculated the Euclidean distance between the target data and the output of a multilayer Artificial Neural Network (ANN) trained with undamaged structure data. They proposed an unsupervised learning approach to locate damaged structures from the increased Euclidean distance. Truong et al. [9] introduced deep feedforward neural networks (DFNN) to detect damage to truss structures. They simulated damaged structures by reducing the elastic modulus of individual elements and verified the performance of the proposed DFNN. Changa et al. [10] estimated damage locations and severity by training neural networks with modal properties after reducing stiffness to create damage patterns. Abdeljaber et al. [11] proposed a one-dimensional CNN that extracts features from raw accelerometer signals and classifies damage. With the development of computer vision, 2D CNN has been successfully used as a vision-based SHM technique [12]. CNNs trained with structure images successfully classify surface damage such as concrete cracks and spalling conditions [13,14,15,16].

In this paper, we propose a Graph Neural Network (GNN) to evaluate the cable cross-sectional area reduction caused by corrosion or fracture of structures. The proposed method consolidates the overall structure and geometric features of the cable-stayed bridge. The deep learning-based damage detection method requires sufficient data with various damaged states for neural network learning. However, it is almost impossible to obtain data on damaged bridges in operation for safety reasons. Besides, to learn a classifier to detect a damaged location, data on each damaged location is required. Moreover, it is impossible to obtain balanced data for all damaged cases because in the real world, the damage scenarios are very rare since the bridge must guarantee a safe condition for long service life [6]. Therefore, it is difficult to train a damage detection model due to the difficulty of collecting data and the class imbalance problem. To resolve these limitations, there is a growing need for research on applying SHM technology to digital twin models. Therefore, we introduce a Practical Advanced Analysis Program (PAAP) [17,18,19,20] to extract the GNN training data. PAAP is very efficient as it can capture material non-linearities of space structures. In addition, the reliability of PAAP has been evaluated for the cable-stayed bridges [20,21] and suspension bridges [22,23].Therefore, it is possible to simulate cable-stayed bridges with various conditions, such as material properties and loads, similar to real-world bridge-like conditions. Furthermore, we can extract data on various damage states of cable-stayed bridges that cannot be obtained in real-world bridges and use them for deep learning model training. Besides, we can predict the real-world bridge state by utilizing real SHM data into the trained deep learning model. In this work, we employ PAAP to analyze the cable tensions of cable-stayed bridge models with reduced cable areas. Moreover, we represent the sensor data as a graph composed of vertices and edges using the generated tension data and spatial information. Studies using point clouds produced by laser scanning have been proposed to evaluate the structure state regarding the entire bridge structure and 3D spatial information data between sensors [24,25,26]. We notice that 3D spatial information of bridges can provide helpful information about the structure states from previous studies. However, using point cloud data for structural health evaluation is possible only for visible elements such as cracks and spalling [27,28], and the point cloud data are not directly related to the loss of stiffness or strength since the point cloud data do not adequately capture the depth due to occlusion [29]. Since GNN can learn the graph-structured data, it resolves the limitation of CNN, which accepts only the grid-structured data. Thanks to the rapid development of GNN, which is capable of recent graph prediction, the utilization of deep learning increases in various domains such as traffic forecasting [30,31], recommendation system [32,33], molecular property prediction [34], and natural language processing [35,36]. Recently, a study using GNN for cable-stayed bridge monitoring has been proposed. Li et al. [37] explored the spatiotemporal correlation of the sensor network in a cable-stayed bridge using the graph convolutional network and a one-dimensional convolutional neural network. They showed that the proposed method effectively detects sensor faults and structural variation. We expect GNN to be actively examined as an SHM technology in the future. In this study, we use Message Passing Neural Network (MPNN), a representative architecture designed to process graph data. Glimer et al. [34] proposed the MPNN, a GNN framework that represents the message transfer between the vertices of the graph as a learnable function. MPNN learns the representation of the graph while repeating a vertex update with messages received from neighboring vertices. We create a graph with the connection relationship between the cable-stayed bridge nodes and apply the node and element data as the vertex features and edge features of the graph, respectively. We train MPNN to estimate damaged cables using the graphed sensor data. We also estimate the cross-sectional area of the damaged cable and identify the damaged cable location to reveal a detailed bridge condition. We adopt a multi-task learning method to secure that our deep learning model predicts two tasks effectively. The multi-task learning benefits while learning related tasks together [38]. Since estimating the location and cross-sectional area of damaged cables are not independent tasks, deep learning models can be optimized efficiently while simultaneously learning both tasks.

## 2. Background

Structural health conditions of cable-stayed bridges are generally monitored based on cable tension changes related to cable area parameters. The tensile forces on cables inevitably change when one or more cables are damaged. A machine learning model is one of the damage detection techniques that identify damage location and degree. This section presents a fundamental understanding of the cable-stayed bridge model and our proposed approach for damage detection. A robust structural analysis program, Practical Advanced Analysis Program (PAAP), is introduced, followed by our cable-stayed bridge model. We then introduce a deep learning theory to understand Message Passing Neural Network (MPNN) adopted as a damage detection technique in this work.

### 2.1. Practical Advanced Analysis Program (PAAP)

The PAAP is an efficient program in capturing the geometric and material non-linearities of space structures using both the stability function and refined plastic hinge concept. The Generalized Displacement Control (GDC) technique is adopted for solving the nonlinear equilibrium equations with an incremental-iterative scheme. This algorithm accurately traces the equilibrium path of the nonlinear problem with multiple limit points and snap-back points. The details of the GDC are presented in [17,39]. In many studies of cable-stayed bridges [21,40], cables have been modeled as truss elements, while pylons, girders, and cross-beams were modeled as plastic-hinge beam-column elements. The plastic-hinge beam-column elements utilize stability functions [41] to predict the second-order effects. The inelastic behavior of the elements is also captured with the refined plastic hinge model [42,43]. To correctly model the realistic behaviors of cable structures, the catenary cable element is employed in the PAAP due to its precise numerical expressions [40]. The advantage of the PAAP is that the nonlinear structural responses are accurately obtained with only one or two elements per structural member leading to low computational costs [17,21]. Thus, the PAAP is employed to analyze and determine the cable tensions in our cable-stayed bridge model.

### 2.2. Cable-Stayed Bridge Model

**Figure 1:**
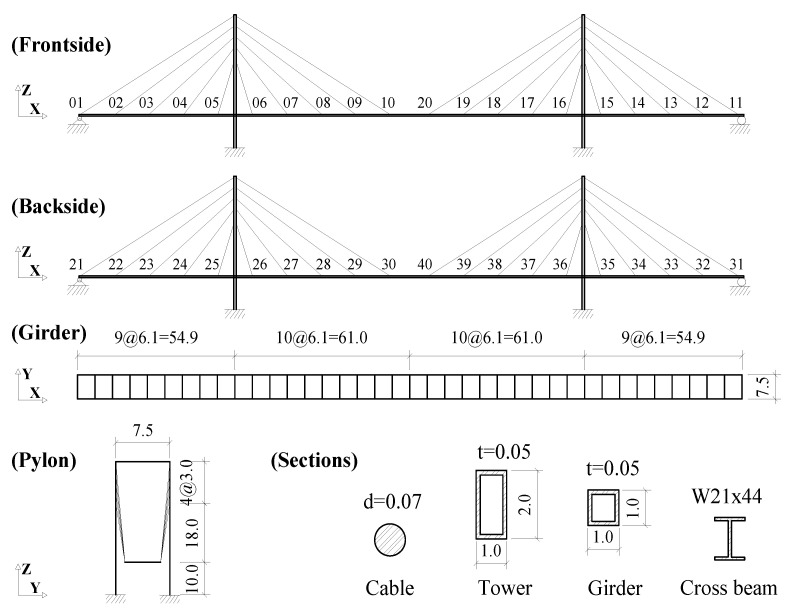
Cable-stayed bridge model in this study (unit: m).

A cable-stayed bridge model of the semi-harp type is proposed as shown in Figure 1. The bridge has a center span of 122 m and two side spans of 54.9 m. Two 30 m-high towers support two traffic lanes with an overall width of 7.5 m. Pylons, girders, and cross beams are made of steel with a specific weight of 76.82 kN/m${}^{3}$. The specific weight of the stayed cable is 60.5 kN/m${}^{3}$. In the PAAP, the girders, pylons, and cross beams are modeled as plastic-hinge beam-column elements. The stayed cables are modeled as catenary elements. For simplicity in determining the damage of the cable, only the dead load induced by the self-weight of the bridge is considered.

### 2.3. Multilayer Percpetron

The most straightforward neural network, Multilayer Percpetron (MLP), has a structure that includes multiple hidden layers between the input layer and the output layer. In each fully-connected hidden layer, the activation function is applied to the affine function of hidden unit $h^{\left(i\right)}$ as follows.

(1) $$h^{\left(i\right)}=\sigma_{i}\left(\sum_{k}w_{k}^{\left(i\right)}h_{k}^{\left(i-1\right)}+b^{\left(i\right)}\right),$$

where, $w^{\left(i\right)}$ and $b^{\left(i\right)}$ represent the $i^{th}$ hidden layer weight and bias, respectively. σ is an activation function for nonlinear learning. There are various activation functions, and mainly Rectified Linear Unit (ReLU), hyperbolic tangent (tanh), and sigmoid functions defined below are applied frequently.

(2) $$\mathrm{ReLU}\left(x\right)=\max\left(x,0\right)$$

(3) $$\tan h\left(x\right)=\frac{1-\exp\left(-2x\right)}{1+\exp\left(-2x\right)}$$

(4) $$\mathrm{sigmoid}\left(x\right)=\frac{1}{1+\exp\left(-x\right)}.$$

Dropout is applied to the hidden layer to prevent overfitting of the neural network. During the training process, the dropout disconnects randomly selected hidden units at a certain probability, such as the dropout rate. Then, the network becomes more robust because the network output does not depend only on a specific unit.

### 2.4. Recurrent Neural Network

**Figure 2:**
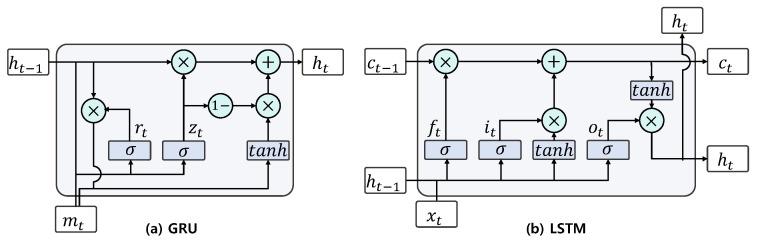
(**a**) GRU structure and (**b**) LSTM structure.

Recurrent Neural Network (RNN) generates the output with current input and hidden state representing past information of sequence data. Typical RNN structures, Gated Recurrent Units (GRUs) [44] and Long Short-Term Memory (LSTM), support the gating of the hidden state and control information flow. Figure 2a shows how the hidden state is calculated in GRU. GRU computes the reset gate $r_{t}\in R^{k}$ that controls the memory from $i^{th}$ data $m_{i}\in R^{d}$, where *d* is the dimension of $m_{i}$, in the input sequence and the update gate $z_{t}\in R^{k}$, where *k* is the dimension of the hidden state, that controls the similarity between the new state and the old state. GRU integrates the computed gates to determine the candidate hidden state ${\tilde{h}}_{t}\in R^{k}$ and the hidden state $h_{t}\in R^{k}$. The equations of GRU are as follows.

(5) $$r_{t}=\sigma \left(W_{mr}m_{t}+W_{hr}h_{t-1}+b_{r}\right)$$

(6) $$z_{t}=\sigma \left(W_{mz}m_{t}+W_{hz}h_{t-1}+b_{z}\right)$$

(7) $${\tilde{h}}_{t}=\tanh\left(W_{mh}m_{t}+r_{t}⊙\left(W_{hh}h_{t-1}\right)+b_{h}\right)$$

(8) $$h_{t}=\left(1-z_{t}\right)⊙{\tilde{h}}_{t}+z_{t}⊙h_{t-1},$$

where, ⊙ and σ are Hadamard product and sigmoid functions, respectively. $W_{mr},W_{mz},W_{mh}\in R^{d\times k}$ and $W_{hr},W_{hz},W_{hh}\in R^{k\times k}$ are weight parameters. $b_{r},b_{z},b_{h}\in R^{k}$ are biases.

Figure 2b shows the computation process of hidden state in LSTM. The cell state $c_{t}\in R^{k}$ and hidden state $h_{t}\in R^{k}$ for input data $x_{t}\in R^{d}$ with input gate $i_{t}$, forget gate $f_{t}$, output gate $o_{t}\in R^{k}$ are computed as follows.

(9) $$i_{t}=\sigma \left(W_{xi}x_{t}+W_{hi}h_{t-1}+b_{i}\right)$$

(10) $$f_{t}=\sigma \left(W_{xf}x_{t}+W_{hf}h_{t-1}+b_{f}\right)$$

(11) $$o_{t}=\sigma \left(W_{xo}x_{t}+W_{ho}h_{t-1}+b_{o}\right)$$

(12) $$c_{t}=f_{t}⊙c_{t-1}+i_{t}⊙tanh\left(W_{xc}x_{t}+W_{hc}h_{t-1}+b_{c}\right)$$

(13) $$h_{t}=o_{t}⊙\tan h\left(c_{t}\right),$$

where $W_{xi},W_{xf},W_{xo},W_{xc}\in R^{d\times k}$ and $W_{hi},W_{hf},W_{ho},W_{hc}\in R^{k\times k}$ are weight parameters. $b_{i},b_{f},b_{o},b_{c}\in R^{k}$ are biases.

The set2set model [45] is permutation invariant for input data using an attention mechanism.

(14) $$q_{t}=LSTM\left(q_{t-1}^{*}\right)$$

(15) $$e_{i,t}=f\left(m_{i},q_{t}\right)$$

(16) $$a_{i,t}=\frac{exp\left(e_{i,t}\right)}{\sum_{j}exp\left(e_{j,t}\right)}$$

(17) $$r_{t}=\sum_{i}a_{i,t}m_{i}$$

(18) $$q_{t}^{*}=q_{t}r_{t},$$

where $m_{i}$ is the memory vector, $q_{t}$ is the query vector, and *f* is the dot product.

### 2.5. Message Passing Neural Network

We assess the damage of the bridge structure using Graph Neural Network (GNN) to apply the sensor network topology. GNN is a powerful deep learning model that manipulates graph-structured data, and it is recently adopted in various domains. GNN updates the hidden state of the vertex with the neighbor information, captures the hidden patterns of the graph. Moreover, it effectively analyzes and infers the graph. MPNN [34] is a general framework of GNN. It has been employed to evaluate chemical properties by representing 3D molecular geometry as a graph.

**Figure 3:**
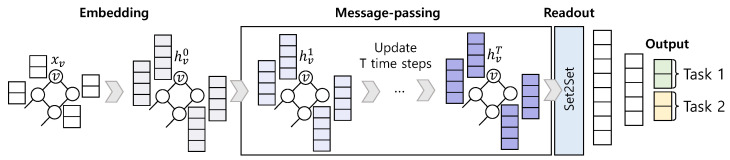
MPNN architecture.

Graph, *G*, consists of a vertex set, *V*, and an edge set, *E*. We denote the feature of vertex, v∈V, as $x_{v}$ and the feature of edge, (u,v)∈E, as $e_{uv}$. As shown in Figure 3, MPNN processes the embedded vertices into a message-passing step and a readout step.

In the message-passing step, each vertex receives the aggregated message $m_{v}^{t+1}$ from the adjacent vertices along the edges with the message function, $M_{t}$. The hidden state of each vertex is updated with the received message, and the previous state of the vertex is updated with the update function, $U_{t}$. The message passing step is repeated *T* times until the message is delivered to a wider range in the graph. In this study, the $t^{th}$ message function $M_{t}$ and the update function $U_{t}$ are defined as follows.

(19) $$M_{t}\left(h_{v}^{t},h_{u}^{t},e_{uv}\right)=\sigma \left(A\left(e_{uv}\right)\;h_{u}^{t}\right)$$

(20) $$m_{v}^{t+1}=\sum_{u\in N(v)}M_{t}\left(h_{v}^{t},h_{u}^{t},e_{uv}\right)$$

(21) $$U_{t}=\mathrm{GRU}\left(h_{v}^{t},m_{v}^{t+1}\right),$$

where σ is the ReLU activation function. A(·) is a two-layer neural network generating a matrix and consists of a layer with 2k neurons and ReLU activation, and a layer with k×k neurons. The neighbors of a vertex, $N\left(v\right)=\left\{u\in V|\left(u,v\right)\in E\right\}$, are adjacent vertices connected through edges from *v*. $h_{v}^{t}$ and $h_{u}^{t}$ are the $t^{th}$ hidden states of vertices, *v* and *u*, respectively. The initial hidden state, $h_{v}^{0}$, is the embedding of vertex *v* obtained by substituting $x_{v}$ into the differentiable function. In Equation (21), we define the update function $U_{t}$ as GRU described in Section 2.4. GRU integrates the state of the vertex itself and the message received as $M_{t}$ from adjacent vertices Finally, the hidden state of the $t^{th}$ updated vertex *v* is defined as follows.

(22) $$h_{v}^{t+1}=U_{t}\left(h_{v}^{t},m_{v}^{t+1}\right),$$

The readout step aggregates the last hidden states, $h^{T}$, after iterating the message passing T times. The prediction, $\hat{y}$, for the target data is calculated with the readout function, *R*, as follows.

(23) $$\hat{y}=R\left(\left\{h_{v}^{T}|v\in V\right\}\right).$$

We define the readout function *R* as the set2set model presented in Section 2.4. Since the set2set is invariant for graph isomorphism, it effectively integrates the vertices of the graph and produces a graph level embedding.

## 3. Data Generating Procedure

In this section, we describe how the cable-stayed bridge data used for the MPNN training is generated.The cable damage model is presented based on the elemental area reduction parameter before the measured cables are specified. Then, the structural analyses are performed to analyze the proposed model for reliable datasets that are essential to construct the machine learning model later.

### 3.1. Cable Damage Model

During the service life of cable-stayed bridges, cables are the most critical load-bearing components [46,47]. Thus, the potential damage of cables should be identified early to prevent terrible disasters [48,49]. In this study, the damage of cable-stayed bridges is assumed to be caused solely by the cable damages. In the cable-stayed bridge model, there are a total of 40 cables corresponding to the 40 catenary elements that are numbered as shown in Figure 1. The cable element is supposed to be perfectly flexible [40] with the self-weight distributed along its length. It has a uniform cross-sectional area of 3846.5 mm${}^{2}$ in the intact state of the bridge. The cable damage is expressed through a scalar area reduction variable α with the value between 0 and 1 as follows:

(24) $$A_{d}=\left(1-\alpha \right)\cdot A_{i}$$

where $A_{i}$ represents the cross-section area of the cable in the intact state and $A_{d}$ denotes the cross-section area of the cable in the damaged state. α is the elemental area reduction parameter to be identified. It is noted that α=1 indicates a destroyed cable, and α=0 indicates an intact cable.

### 3.2. Observed Cables

**Figure 4:**
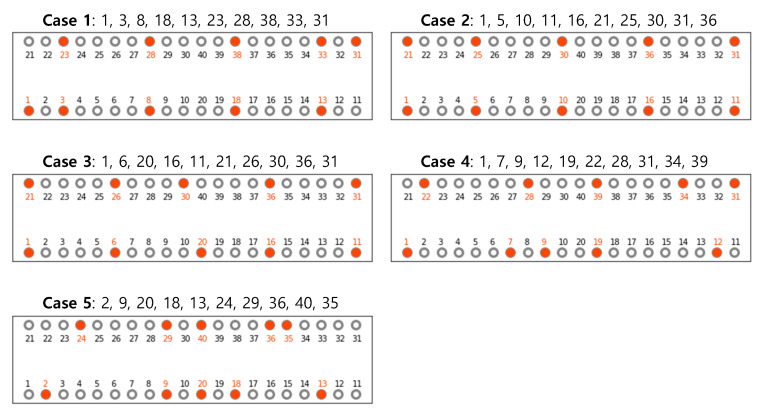
5 sensor layout cases.

In most structural health monitoring systems of cable-stayed bridges, sensors are installed to collect data from specific cables due to the cost-effectiveness. The quantity of surveyed cables depends on the scale and complexity of the bridge and the monitoring objectives [47,50]. At surveyed locations, cable sensitivity and safety degrees are evaluated. The measured data is automatically observed and stored as essential sources for later usage during the monitoring time. In this study, 10 out of 40 cables are surveyed, including 5 cables on the front side and 5 cables on the backside. We examine five sensor layout cases as shown in Figure 4. However, we do not include optimization of sensor placement (OSP) in the scope of this study. Since we do not apply the OSP technology, the sensors are evenly arranged. We analyze multiple cases to avoid skewing the experimental results to specific cases. The tensile forces within these cables are determined by simulating the proposed model in the PAAP. Using the GDC method [20,51] to solve nonlinear problems, the PAAP divides the dead load into many incremental steps. Obtained results from the structural system at each incremental step, including internal forces, deformations, displacements, etc., are exported and stored in data files. However, only cable tensions at the step corresponding to the bridge self-weight are considered as measured data.

### 3.3. Generating Data

Different cable-stayed bridge models are constructed and analyzed by using the PAAP. The geometry configurations of the bridge girders, pylons, and cross beams are kept constant, while only the cable cross-sectional areas vary. The output is the tensile force on observed cables as determined in Section 3.2. The complete procedure for generating data is presented in Table 1. For single-cable damage, 4000 data samples are generated as the elemental area reduction parameter varies from 0 to 1 with a step of 0.01. To evaluate the cable system failure based on the simulation results, the prediction model performs a reverse problem. The tensile forces of 10 observed cables are examined as the input, while the predefined elemental area reduction parameters are employed for the target data. These input and target data are utilized for the training and validation of the proposed damage detection model concerning the cable-stayed bridge. Upon completion, the model predicts damaged cable and its cross-section area $A_{d}$ according to 10 inputs of cable tensile forces $S=\left[T_{1},T_{2},T_{3},\ldots ,T_{10}\right]$.

## 4. Proposed Method for Damage Assessment

In this section, we describe MPNN for damage assessment of the cable-stayed bridge. We present the specific MPNN configuration and show how to apply the proposed multi-task learning to identify the location of the damaged cable created in Section 3 and the cross-sectional area of the corresponding cable.

### 4.1. Configuration of the Proposed Network

We define the graph vertex feature, $x_{v}$, with tensile forces of the 10 cables. The edge feature $e_{uv}$ is defined as the thresholded Gaussian kernel [52] as presented in the equation below using the XYZ coordinates of the nodes on the girder connected to the 10 cables.

(25) $$edge_{uv}=\left\{\begin{array}{cc} \exp\left(-\frac{\mathrm{dist}{\left(u,v\right)}^{2}}{\sigma^{2}}\right), & \mathrm{if}\;\mathrm{dist}\left(u,v\right)\;<\gamma \\ 0, & \;\mathrm{otherwise}\;, \end{array}\right.$$

where we set the threshold γ to 0.1 and σ is the standard deviation of distances. Since we only define vertices and edges as tension and distance, respectively, the dimensions of vertex and edge features are all 1. We embed the vertex features $x_{v}$ representing the tensor into single fully connected hidden layers with the ReLU activation function. The embedded vertex state is updated with the message function $M_{t}$ in Equation (19), and the update function $U_{t}$ in Equation (21). The hyperparameters of the network we tune in the message-passing step include the vertex embedding dimension, the number of iterations of the message passing step, and the hidden state dimension. Also, we tune the number of LSTM layers of the set2set model for the global pooling, readout function *R*, and the number of computations, which is another hyperparameter of the set2set model. We add the fully connected hidden layer with the ReLU activation function with the same number of neurons as the vertex embedding. The predictions for target data are generated in two output layers, each of 20 linear units. We describe the two outputs in the next section.

### 4.2. Multi-Task Learning on MPNN

The target data to determine the cable health of the cable-stayed bridge are the damaged cable location and the damage degree (i.e., cross-sectional area, $A_{d}$). Therefore, we adopt multi-task learning to make MPNN learn two tasks effectively. The advantage of multi-task learning is that by predicting multiple tasks simultaneously, related tasks could be learned more efficiently. Therefore, learning to predict the cross-sectional area of the damaged cable and learning to classify the damaged cable simultaneously improves learning efficiency. As shown in Figure 3, the proposed MPNN has outputs for task1 and task2, which are the classification of the damaged cable and the prediction of the cross-sectional area of the damaged cable, respectively. The first task is classification, and the second task is a prediction on continuous data (i.e., regression). Therefore, we utilize the cross-entropy loss function for task1 and the mean absolute error loss function for task2 defined as follows.

(26) $$L_{\mathrm{task}1}=-\sum_{i}D_{i}\;\log\frac{\exp\left(\hat{D_{i}}\right)}{\sum_{j}\exp\left(\hat{D_{j}}\right)}$$

(27) $$\begin{array}{c} L_{\mathrm{task}2}=\left|A_{d}-\hat{A}\cdot M\right|, \end{array}$$

where $D_{i}$ represents the target for *i*th label for the case that the *i*th cable is damaged. $A_{d}$ is the target for the cross-sectional area of a single damaged cable in the range, 0.99 to 0.0. $\hat{A}\in R^{40}$ is the vector output by the network for the second task. We define mask $M\in R^{40}$ as a vector in which one element corresponding to the index of the damaged cable is 1, and all others are 0. In the training phase, the position of 1 in the mask M is actually the index of the damaged cable. $L_{\mathrm{task}2}$ is actually the error between the cross-sectional area of the damaged cable, $A_{d}$, and the dot product of $\hat{A}$ and M. Therefore, the loss for task2 is actually only calculated on the damaged cable. In the test step, the mask M is created as an output for the network classification. Then $\hat{A}\cdot M$ means the estimated cross-sectional area of the cable that the network classified as damaged. We define the total loss $L_{\mathrm{total}}$ by combining $L_{\mathrm{task}1}$ and $L_{\mathrm{task}2}$ as follows.

(28) $$\begin{array}{c} L_{\mathrm{total}}=L_{\mathrm{task}1}+\frac{1}{{∥M∥}_{1}}L_{\mathrm{task}2}. \end{array}$$

The total loss $L_{\mathrm{total}}$ is the sum of task1 (classification) and task2 (regression) scaled by L1-norm of mask M.

## 5. Performance Evaluation

To evaluate the proposed model introduced in Section 4, we generate data with the cable-stayed bridge having damaged cables through the PAAP described in Section 3. We preprocess the generated data for the model training and optimize the MPNN model. Then, we train the proposed MPNN to validate the prediction outcomes. We also train the MLP and compare it with the MPNN model results. The input data of the MLP is only ten cable tension data. MLP has four hidden layers with ReLU activation, and we have added a dropout layer to each hidden layer. Similar to MPNN, the MLP output layer generates 80 predictions for two tasks. Additionally, we compare the results with ones by the machine learning technique, XGBoost. Also, we compare the multi-task learning with a network performing only one task. The number of network outputs for the damaged cable classification, which is task1, is 40 and the loss function is the cross-entropy shown in Equation (26). Furthermore, the number of network outputs for the area estimation of damaged cables, which is task2, is 1, and the loss function is the mean absolute error presented in Equation (27).

### 5.1. Data Preprocessing and Optimization

As mentioned in Section 3, we generate the data for 4000 cases. The input data is the cable-stayed bridge data represented as a graph, as described in Section 4, and the target data include the index and its cross-sectional area of the damaged cable labeled between 1 and 40. The cross-sectional area of the damaged cable is (1−α), which is between 0.0 (broken state) and 0.99. The α is an elemental area reduction parameter defined in Section 3. We scale the vertex feature values, tensile forces, between 0 and 1, as presented as follows.

(29) $$T^{\prime }=\frac{T-\min T}{\max T-\min T}$$

We divide the data into 6:1:3 and generate a 2400 training set, 400 validation set, and 1200 test set.

Table 2 presents the ranges of hyperparameters and selected optimal values for each model. We select the best hyperparameters in the validation set using Tree-structured Parzen Estimators (TPE) [53] with 20 trials. Moreover, we terminate trials with poor performance using Asynchronous Successive Halving Algorithm (ASHA) [54]. We specify the hyperparameters of MPNN in Section 4.1. The hyperparameters of MLP are the number of hidden neurons in each layer and the dropout rate. We optimize the hyperparameters that determine the network structure, batch size, and learning rate. We perform the hyperparameter optimization individually for each of the 5 cases and models.

We utilize the ADAM optimizer [55] and train the MPNN model to minimize the loss function, which is defined in Equation (28). We set the number of epochs to 1000. Then we decay by multiplying the learning rate decided from the hyperparameter optimization by 0.995 per epoch. We use Pytorch and Deep Graph Library (DGL) on a single NVIDIA Geforce RTX2080Ti GPU for network implementation and optimization. We train the MLP model with the same settings as the MPNN model.

### 5.2. Results

In this section, we report the results of the deep learning network for the test set. We examine the accuracy to evaluate the damaged cable classification performance. We employ the mean absolute error (MAE), the root mean squared error (RMSE), and the correlation coefficient between target data and output data as measures to compare the cross-sectional area prediction. MAE, RMSE, and correlation coefficient are defined as follows.

(30) $$\mathrm{MAE}=\frac{\sum_{i}^{n}\left|y-{\hat{y}}_{i}\right|}{n}$$

(31) $$\mathrm{RMSE}=\sqrt{\frac{\sum_{i}^{n}{\left(y-{\hat{y}}_{i}\right)}^{2}}{n}}$$

(32) $$\mathrm{Correlation}\;\mathrm{coefficient}=\frac{\sum_{i}^{n}\left(y_{i}-\bar{y}\right)\left({\hat{y}}_{i}-\bar{\hat{y}}\right)}{\sqrt{\sum_{i}^{n}{\left(y_{i}-\bar{y}\right)}^{2}}\sqrt{\sum_{i}^{n}{\left({\hat{y}}_{i}-\bar{\hat{y}}\right)}^{2}}},$$

where *n* is the number of samples, and *y*, $\hat{y}$, $\bar{y}$, and $\bar{\hat{y}}$ are the target, output, and the average of the target, and the average of the output, respectively. The lower the MAE and RMSE and the higher the correlation coefficient, the better the performance.

Table 3 summarizes the results of MPNN, MLP, and XGBoost. When comparing MPNN with MLP and XGBoost, the classification accuracy and correlation are always higher, and the error for cross-sectional area estimation is lower. Besides, the classification accuracy of MLP drops to 93.58%, depending on the sensor layouts. However, MPNN is more stable with an accuracy of over 98.33% in all 5 cases. Also, for the cross-sectional area prediction, MPNN is better and more stable than MLP and XGBoost. Meanwhile, the multi-task learning performance is similar to one of the single-task learning in which each task is individually trained. However, when multi-task learning is applied, we need to train the network only once, whereas training the network with the single-task increases the time cost by the number of tasks. Therefore, multi-task learning is efficient because it learns multiple tasks simultaneously while achieving performance similar to learning single tasks.

**Figure 5:**
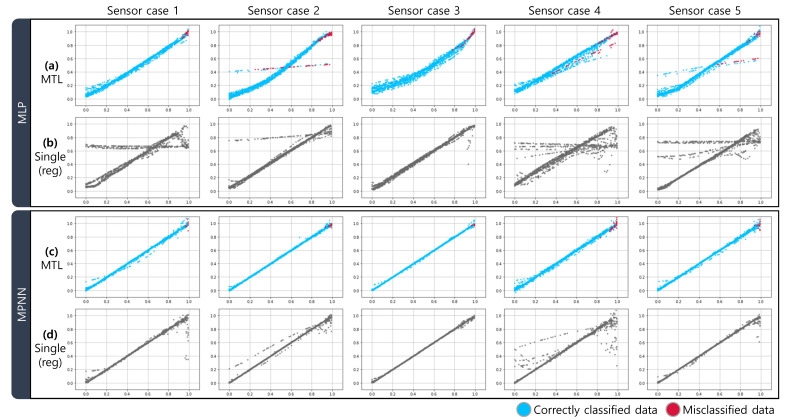
Scatter plots of MLP and MPNN on the test set for estimating the cross-sectional areas of damaged cables in 5 cases. The x-axis is the actual cross-sectional area (target data), and the y-axis is the predicted cross-sectional area. Correctly classified data are indicated as blue points, and incorrectly classified data are shown as red points in multi-task learning. (**a**) MLP with multi-task learning, (**b**) MLP with single-task learning (regression), (**c**) MPNN with multi-task learning, (**d**) MPNN with single-task learning (regression).

Figure 5 shows scatter plots showing the relationships between the predicted values and actual values for the cross-sectional area estimation of the damaged cables. As shown in Figure 5a,b, which are the results of MLP, since many points deviate enormously from the straight line, especially in cases 2, 4, and 5, we confirm that the errors in the prediction of the cross-sectional area are considerable. However, in the scatter plots of MPNN shown in Figure 5c,d, the data points are closer to the straight line than MLP for all cases. For the classification analysis, in the multi-task learning results Figure 5a,c, we confirm that the red points, which are misclassified data, are mainly concentrated when the cross-sectional area is close to 1. It appears that the smaller the damage, the more likely the damaged cable will be misclassified.

**Figure 6:**
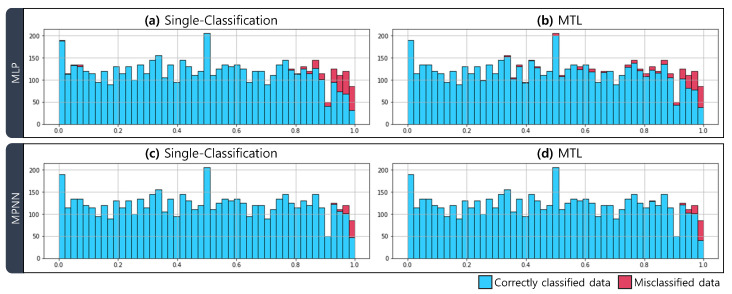
Histograms of correctly classified data (blue) and misclassified data (red) according to the damaged cable cross-sectional areas. (**a**) MLP with single-task learning (classification), (**b**) MLP with multi-task learning, (**c**) MPNN with single-task learning (classification), (**d**) MPNN with multi-task learning are presented.

Figure 6 shows the histogram of correctly classified data and incorrectly classified data for varying cross-sectional areas. We observe that in all four network results, in general, the correctly classified data (blue) are evenly distributed, and the misclassified data (red) are skewed toward the cross-sectional area close to 1. For more accurate verification, we divide the cross-sectional area range by 0.1 and calculate the classification accuracy of the data included in each range.

Table 4 presents the accuracies according to the cross-sectional areas. When the cross-sectional area is less than 0.9, the accuracy of the MLP and XGBoost> is between 81% and 100%. When the cross-sectional area is more than 0.9, the classification performance of MLP drops to 50%, and the best accuracy is 79.59% in case 5. However, when the cross-sectional area of MPNN is less than 0.9, the accuracy is over 99.2%. Also, in both multi-task learning and single-task learning, the accuracy of all cases is almost 100%. When the cross-sectional area is more than 0.9, the accuracy of MPNN is at least 73.47% and at most 91.84%. When the cable cross-sectional area loss is small, the accuracy of MPNN decreases slightly, but we notice that MPNN classifies damaged cables relatively more reliably than MLP and XGBoost. Besides, it is noticed that for each cross-sectional area change, none of the multi-task learning method and the single task learning method always outperforms in all cases.

**Figure 7:**
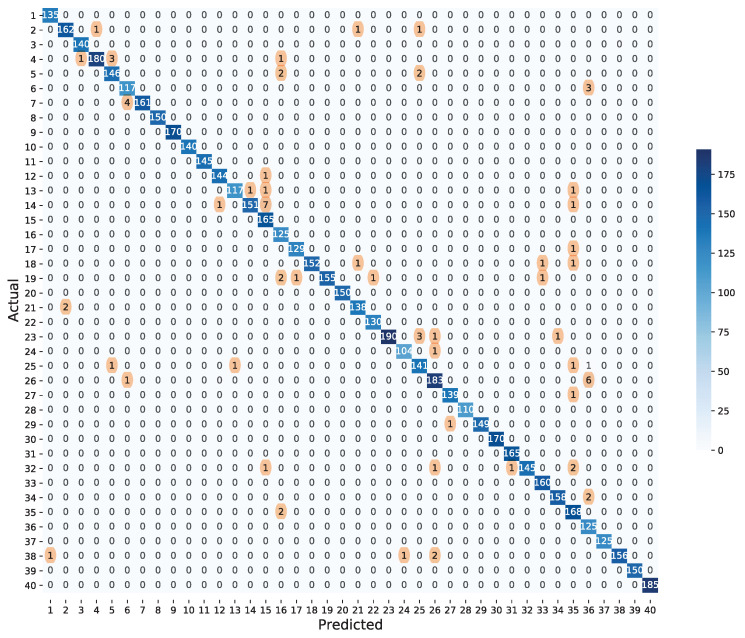
Confusion matrix.

Figure 7 shows the confusion matrix of MPNN combining all 5 cases. Since there are a few misclassified data, we highlight the misclassified data with the orange shade. We observe that the location of the misclassified cable tends to be close to the damaged cable. For example, when the actual labels are 4, 7, 13, and 14, the predicted labels are 3, 6, 14, 16, and 15, respectively. These cables are located next to each other.

**Figure 8:**
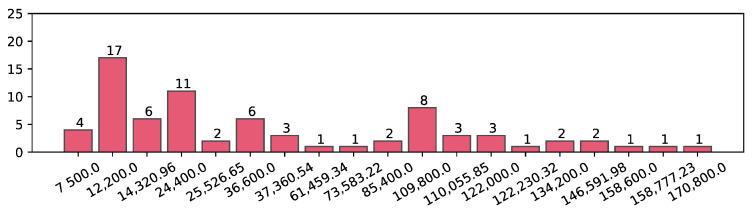
Histogram of the distance between the original damaged cable and misclassified cable in the misclassified data is presented.

Figure 8 shows a histogram of the sensor distances corresponding to the actual damaged cable and the cable incorrectly classified by the network for all 5 cases to illustrate the spatial relationship between the actual labels and the predicted labels in more detail. Of 75 incorrectly classified data, the distance between 17 actual damaged cables and predicted damaged cables is only 12,200, which is the distance between adjacent cables. Therefore, if we apply the proposed method to an actual bridge, we urge that the cables on both sides of the classified cables must be checked to avoid more significant damage.

The tensions used as input data are measured only on ten cables. However, the proposed technique assesses damages to all 40 cables. Therefore, we need to compare the predictions for the ten cables with tension data and the 30 cables without tension data. Table 5 shows the results for the damaged cables with sensors and the damaged cable without sensors in all cases.

We notice that the performance of MPNN is more remarkably different than that of MLP and XGBoost when the cable with no sensor attached is damaged. When the cable with the sensor is damaged, the classification accuracy is higher compared to the case that the cable without the sensor is damaged. The regression performance also shows the similar pattern except for case 1, 2, and 3. In case 1, 2, and 3, on the contrary, the result is better when the cable without the sensor is damaged in results of MLP and MPNN. This seems to be related to a problem with the sensor position. As seen in Figure 4, unlike case 4 and case 5, the spacing of sensors in cases 1, 2, and 3 is always less than five cables. In case 1, 2, and 3, since the sensors are evenly distributed, we observe that even if the cable without the sensor is damaged, especially in the regression task, the performance degradation does not appear. Table 6 for more detailed results for case 3 shows the classification result (accuracy, precision, recall, and F1 score) and the regression result (MAE, RMSE, and correlation coefficient) for each cable of the cross-sectional area when the cable is damaged.

The accuracy, precision, recall, and F1 score are calculated as follows.

(33) accuracy=(TP+TN)/(TP+TN+FP+FN)

(34) precision=(TP)/(TP+FP)

(35) recall=(TP)/(TP+FN)

(36) F1=(2TP)/(2TP+FP+FN),

where TN is true negative, TP true positive, FN false negative, and FP false positive. The rows shaded in green indicate ten cables with tension data, and all four measures for the classification, including accuracy, precision, recall, and F1 score, are 1.00. For each measure, values in the lower 5% of performance appear in red, and values in the upper 5% of performance appear in blue. Then we observe that cable 25 and cable 33 have the lowest precisions, which can be interpreted as a relatively high probability of misclassification among those predicted by MPNN that cable 15 and cable 24 are damaged in multi-task learning. Cable 14 has the lowest recall in both multi-task learning and single-task learning. Therefore, we can interpret that when cable 14 is damaged, MPNN is relatively likely to predict that the other cable is damaged. Also, the accuracy and F1 score of cable 14 and cable 15 are the lowest in multi-task learning. In addition to this, cable 14 in both multi-task learning and single-task learning have lower performance metric values for the classification. The cables mentioned so far are all sensorless cables in case 3. Unlike classification, the cross-sectional area prediction seems to be mostly unrelated to the use of tension data. For example, the regression performance is excellent even for the damaged cable 17 and 9 that do not have any tension data. Therefore, estimating the cross-sectional area of a single damaged cable is less related to the tension data-position than the classification.

### 5.3. Discussion

We have shown that MPNN can successfully assess cable damage estimation and outperform MLP. When the cable cross-section is damaged less than 0.9, MPNN always classifies the damaged cable more accurately than MLP. However, when the cable cross-section area damage is negligible as 0.9 or more, the classification accuracy slightly deteriorates. Once we improve the deep learning network to work more accurately for the bridge structure data with minor damage, we expect that the overall accuracy becomes 100%. Misclassified cables by MPNN are often located right next to the actual damaged cables. We can utilize these MPNN misclassification trends to update the algorithm and training process. However, since MPNN has reached 98% or even higher accuracy, achieving sufficiently satisfactory results, we believe that MPNN has potential as an SHM technology. Also, the multi-task learning performance is similar to the multiple single-task learning performance. Therefore, we have shown that multi-task learning can efficiently learn a single network that evaluates a bridge state.Besides, we have presented that the multi-task learning technique achieves similar performance to the network learning two tasks while learning only one network. Therefore, it is possible to evaluate the bridge conditions in several ways using only one network. It is worth adding more tasks other than predicting only the cross-sectional area of the cable in the future.

#### 5.3.1. Contribution

We confirmed that MPNN has a higher overall performance than MLP and XGBoost. In particular, MPNN has a significant difference in performance from MLP and XGBoost when a cable without a sensor is damaged. Since MPNN can process spatial information between sensors, it appears that damages to cables without sensors can be estimated more successfully. MPNN has the advantage of being able to transmit information according to the connectivity relationship between sensors through a structure that passes messages. Also, by adding a readout function, MPNN produces an output as a graph unit value from node information, making it possible to predict the state of 40 cables effectively. In this paper, we captured spatial correlation by considering sensor geometry with MPNN. Moreover, we showed that two tasks (classifying damaged cables and estimating cross-sectional area) could be efficiently trained using multi-task learning. Besides, we proposed a loss function using a mask so that the damaged cable could be more successfully estimated.

#### 5.3.2. Limitation

We could represent the sensor geometry as a graph in this study but did not consider mechanical properties such as the material type or Young’s modulus of the structure since it is difficult to define the relationship among the sensors when several types of materials are included between the two sensors. Besides, it may be challenging to learn the entire structure behaviors with the proposed method because it is not possible to deduce the entire topology of the bridge with only a few sensor data, which may necessitate the installation of a sufficiently large number of sensors. However, it cannot always be satisfied due to cost constraints. Therefore, to understand the condition of the entire bridge with only a small number of sensors, we fundamentally need to examine the influence of the sensor locations and apply it to enhance the model.

#### 5.3.3. Extension To Multiple Damaged Cables

In this study, we assess the GNN-based SHM technology assuming that only one cable is damaged. To generate more realistic data similar to a real-world bridge, we need to simulate several damaged cables. We can apply the proposed technique by transforming from one label classification to a multi-label classification problem even when the number of damaged cables is unknown. A straightforward approach is to replace the cross-entropy loss function with a loss function used for multi-label classification, such as binary cross-entropy. However, the threshold for deciding how many cables to classify as damaged should be appropriately set, which is an essential component. If the target data, which indicates the cross-sectional areas of the actual damaged cables, is represented as a vector of dimension w, where w is the number of damaged cables, then we can use the mask as shown in Equation (27) and compute the loss for the regression task. Suppose we multiply the 40 outputs of the network for task2 and the mask that is a 40×w matrix, where each column is a one-hot encoding vector representing the locations of the damaged cables. In that case, we obtain the cross-sectional areas of the damaged cables. Similarly, the mask is created as the actual label in the training step and the predicted label in the test step. The total loss is obtained by combining the loss for the classification task and the loss for the regression task by scaling the L1-norm of the mask. We do not desire to weigh the regression task more than the classification task as the number of damaged cables increases. We can prevent this by scaling the regression loss with the mask. Therefore, even with multiple damaged cables, we can still apply the proposed method as a multi-task learning approach. As discussed above, we will review the conditions for making the cable-stayed bridge model and the real-world bridge similar and improve our technique to apply to the real-world bridge.

## 6. Conclusions

In this paper, we defined the sensor data as a graph composed of vertex and edge features. We proposed a damage assessment method of a cable-stayed bridge applying the graph representation on MPNN. We used tension data of only 10 cables to increase the practicality of the experiment. It is challenging to assess the conditions of all cables with only a limited number of sensors. Nevertheless, MPNN successfully estimated the damage of the cable-stayed bridge. We adopted multi-task learning to enable MPNN to efficiently learn two tasks: to locate damaged cables and predict the cable areas. The performance of MPNN is better than MLP trained for the comparison. MPNN classified damaged cables more reliably than MLP, not only when the cable is completely broken and has a zero area, but also when the damage is relatively small. Therefore, we presume that MPNN can detect damages at an early stage for structural maintenance. Furthermore, we can apply MPNN to actual bridge data when we have material information about the structural components. For example, we can train MPNN with stayed-cable bridge data simulated under the same conditions as real bridges in PAAP and utilize pre-trained MPNN with real bridge data for prediction directly. Additionally, although we simulated only one damaged cable in this study, we will generate data with multiple damaged cables to train the network to consider the more general real-world bridge cases. We also introduced an approach to conduct damage localization and severity assessment with the proposed method when several cables are damaged as a future study. Our model is likely to be extended by applying additional data such as the displacement of nodes and xyz coordinates for vertex features. Moreover, we can further expand the study by training MPNNs to predict structural damages, such as decreased stiffness besides cable conditions.

## Figures and Tables

**Table 1:** Data generating procedure.

***Step 1.***	Input structural geometry, material configurations and set applied loads.
***Step 2.***	Generate M samples $\left(C_{1},C_{2},\ldots ,C_{M}\right)$ of 40 cables of system $C_{i}=\left[A_{1},A_{2},A_{j},\ldots ,A_{40}\right]$, where $A_{j}$ is the cross-section area of damaged cable *j*th in sample *i*th, that is determined as shown Equation (24).
***Step 3.***	Calculate the tension of 10 observed cables $S_{i}=\;\left[T_{1},T_{2},T_{k},\ldots ,T_{10}\right]$ that mentioned in Section 3.2 corresponding to the sample $C_{i}$ using the PAAP, where $T_{k}$ is the measured tension of cable $k^{th}$ in sample $i^{th}$.
***Step 4.***	Save the input and output data to result files.

**Table 2:** Hyperparameter optimization. The optimal values for multitask learning, regression learning, and classification learning are separated by commas and appear in order.

			Optimal Values				
Model	Hyperparameter	Range	Case 1	Case 2	Case 3	Case 4	Case 5
MPNN	Batch size	[16, 32, 64, 128]	16, 16, 16	32, 32, 16	32, 16, 32	16, 16, 16	16, 16, 32
	Learning rate	[0.00001∼0.001]	0.00095, 0.00018, 0.00078	0.00029, 0.00081, 0.00047	0.00070, 0.00043, 0.00076	0.00036, 0.00016, 0.00040	0.00030, 0.00092, 0.00098
	Vertex embedding dim	[8, 16, 32, 64, 128]	32, 128, 128	128, 32, 128	128, 128, 128	64, 128, 64	64, 64, 128
	Hidden state dims	[8, 16, 32, 64, 128]	16, 16, 32	32, 64, 16	8, 8, 16	8, 64, 16	16, 8, 8
	# of message passing steps	[3, 4, 5, 6]	3, 5, 5	6, 4, 3	6, 6, 5	5, 6, 5	4, 4, 3
	# of set2set computations	[1, 2, 3, 4, 5]	5, 2, 1	1, 5, 1	4, 5, 2	1, 4, 1	4, 5, 5
	# of LSTM layers	[1, 2, 3]	2, 3, 3	2, 2, 2	2, 1, 2	3, 1, 2	3, 1, 2
MLP	Batch size	[16, 32, 64, 128]	32, 64, 128	32, 16, 8	16, 128, 16	32, 32, 32	128, 32, 128
	Learning rate	[0.00001∼0.001]	0.00038, 0.00015, 0.00054	0.00003, 0.00044, 0.00029	0.00017, 0.00013, 0.00014	0.00049, 0.00015, 0.00031	0.00025, 0.00044, 0.00072
	# of hidden neurons in the hidden layer 1	[32, 64, 128, 256, 512, 1024, 2048]	1024, 256 1024,	1024, 32, 512	1024, 512, 512	64, 128, 128	2048, 256, 512
	# of hidden neurons in the hidden layer 2	[32, 64, 128, 256, 512, 1024, 2048]	512, 64, 512	2048, 64, 512	256, 2048, 2048	32, 1024, 1024	2048, 128, 512
	# of hidden neurons in the hidden layer 3	[32, 64, 128, 256, 512, 1024, 2048]	2048, 512, 64	2048, 512, 2048	256, 256, 1024	128, 256, 2048	1024, 512, 32
	# of hidden neurons in the hidden layer 4	[32, 64, 128, 256, 512, 1024, 2048]	128, 2048, 512	1024, 32, 128	256, 64, 1024	1024, 128, 64	256, 128, 2048
	Dropout rate	[0.1, 0.2, 0.3, 0.4, 0.5, 0.6, 0.7]	0.1, 0.3, 0.3	0.2, 0.1, 0.1	0.5, 0.2, 0.2	0.1, 0.2, 0.2	0.2, 0.1, 0.1
XGBoost	Minimum sum of instance weight	[1, 5, 10]	1, 5	1, 5	1, 10	1, 1	1, 1
	gamma	[0.5, 1, 1.5, 2, 5]	0.5, 1	2, 1	1, 0.5	2, 0.5	0.5, 0.5
	Subsample ratio of the training instance	[0.6, 0.8, 1.0]	0.6, 1.0	0.8, 1.0	1.0, 1.0	0.6, 1.0	0.8, 0.6
	Subsample ratio of columns when constructing each tree	[0.6, 0.8, 1.0]	0.6, 1.0	0.8, 0.6	0.8, 1.0	1.0, 0.8	0.6, 1.0
	Maximum tree depth	[3, 4, 5]	3, 5	3, 5	3, 5	4, 5	5, 5

**Table 3:** Results of MLP and MPNN on the test set for 5 cases. The best value for each case is noted in bold. (MTL: multi-task learning, Single: single-task learning, Class: classification, Reg: regression).

			Case 1		Case 2		Case 3		Case 4		Case 5	
			MTL	Single	MTL	Single	MTL	Single	MTL	Single	MTL	Single
XGBoost	Class	Acc (%)	-	97.25	-	97.08	-	97.5	-	97.83	-	98.5
		MAE	-	0.1518	-	0.1451	-	0.1422	-	0.1506	-	0.1487
	Reg	RMSE	-	0.1837	-	0.1748	-	0.1713	-	0.1829	-	0.1806
		Corr	-	0.9093	-	0.9253	-	0.9062	-	0.9084	-	0.8815
MLP	Class	Acc (%)	98.33	98.08	93.58	95	97.17	94.17	94	95	97.08	98.08
		MAE	0.0249	0.0832	0.059	0.0369	0.0679	0.0166	0.0492	0.112	0.0408	0.0877
	Reg	RMSE	0.0327	0.1603	0.0827	0.0897	0.0788	0.0314	0.0613	0.1669	0.0611	0.1608
		Corr	0.9953	0.8321	0.9699	0.9541	0.9734	0.9944	0.9885	0.831	0.9807	0.8261
MPNN	Class	Acc (%)	**99.08**	98.83	98.67	**99.17**	**99.33**	99.25	97.75	**98.33**	98.92	**99.17**
		MAE	0.0093	**0.009**	**0.005**	0.008	0.0035	**0.0028**	**0.0104**	0.0265	0.007	**0.0046**
	Reg	RMSE	**0.0331**	0.0433	**0.0121**	0.0282	0.0069	**0.0066**	**0.0175**	0.0843	**0.0138**	0.0199
		Corr	**0.9934**	0.9884	**0.9991**	0.9951	**0.9997**	**0.9997**	**0.9981**	0.9552	**0.9988**	0.9976

**Table 4:** Classification accuracies by cross-sectional areas of damaged cable for 5 cases. For each cross-sectional area, blue indicates the most accurate, and red denotes the least accurate.

		Case 1		Case 2		Case 3		Case 4		Case 5	
	**Area**	**MTL**	**Single**	**MTL**	**Single**	**MTL**	**Single**	**MTL**	**Single**	**MTL**	**Single**
XGBoost	0.00∼0.69	-	99.88	-	99.88	-	**100**	-	**100**	-	**100**
	0.70∼0.79	-	**100**	-	**100**	-	**100**	-	**100**	-	**100**
	0.80∼0.89	-	**100**	-	**100**	-	**100**	-	**100**	-	**100**
	0.90∼0.99	-	**67.35**	-	65.31	-	69.39	-	73.47	-	81.63
MLP	0.00∼0.69	**100**	**100**	97.9	99.42	**100**	99.42	98.36	**100**	99.42	**100**
	0.70∼0.79	**100**	**100**	95.87	99.17	99.17	99.17	95.04	**100**	95.87	**100**
	0.80∼0.89	**100**	**100**	90.4	92.8	96	88	88	84.8	96	99.2
	0.90∼0.99	79.59	76.53	57.14	**54.08**	71.43	**50**	62.24	**58.16**	79.59	**77.55**
MPNN	0.00∼0.69	**100**	**100**	**100**	**100**	**100**	**100**	**100**	**100**	**100**	**100**
	0.70∼0.79	**100**	**100**	**100**	**100**	**100**	**100**	**100**	**100**	**100**	**100**
	0.80∼0.89	**100**	**100**	**100**	**100**	**100**	**100**	99.2	**100**	**100**	**100**
	0.90∼0.99	88.78	85.71	83.67	89.8	91.84	90.82	73.47	79.59	86.73	**89.8**

**Table 5:** MLP and MPNN results for damaged cable with sensor (Y) and damaged cable without sensor (N). The bold indicates the better result between these two.

			Case 1		Case 2		Case 3		Case 4		Case 5	
			Y	N	Y	N	Y	N	Y	N	Y	N
XGBoost	Single-C	Acc (%)	**97.32**	97.23	**97.22**	97.04	**98.29**	97.24	96.98	**98.12**	**98.66**	98.45
		MAE	**0.1462**	0.1536	0.1508	**0.1434**	**0.1359**	0.1443	**0.1489**	0.1512	**0.1437**	0.1503
	Single-R	RMSE	**0.1755**	0.1863	0.1749	**0.1748**	**0.1603**	0.1747	**0.1797**	0.1839	**0.1714**	0.1836
		Corr	**0.9627**	0.8929	**0.9318**	0.9288	**0.9472**	0.8964	**0.9439**	0.8954	**0.9103**	0.8737
MLP	MTL-C	Acc (%)	**100**	97.78	**99.65**	91.67	96.25	**97.46**	**100**	92.02	**100**	96.12
		MAE	**0.0207**	0.0262	**0.059**	**0.059**	0.0756	**0.0654**	0.0496	**0.0491**	**0.0386**	0.0415
	MTL-R	RMSE	**0.0256**	0.0348	**0.0717**	0.0859	0.0874	**0.0758**	**0.059**	0.062	**0.0445**	0.0657
		Corr	**0.9974**	0.9947	**0.9858**	0.9651	0.9634	**0.9766**	**0.9978**	0.9856	**0.9924**	0.977
	Single-C	Acc (%)	**100**	97.45	**98.96**	93.75	**96.93**	93.27	**100**	93.35	**100**	97.45
		MAE	**0.0345**	0.0993	**0.0266**	0.0402	0.0297	**0.0124**	**0.0737**	0.1246	**0.0292**	0.1071
	Single-R	RMSE	**0.0517**	0.1825	**0.0316**	0.1013	0.0554	**0.0177**	**0.0814**	0.1867	**0.0392**	0.1842
		Corr	**0.9935**	0.7773	**0.9992**	0.9398	0.9836	**0.9983**	**0.9937**	0.764	**0.9979**	0.7689
MPNN	MTL-C	Acc (%)	**99.66**	98.89	**99.65**	98.36	**100**	99.12	**99.66**	97.12	**100**	98.56
		MAE	0.0098	**0.0092**	0.0059	**0.0047**	**0.0024**	0.0038	**0.0063**	0.0117	**0.0058**	0.0074
	MTL-R	RMSE	0.057	**0.0196**	0.0201	**0.0081**	**0.0048**	0.0075	**0.0118**	0.019	**0.012**	0.0143
		Corr	0.9823	**0.9977**	0.9976	**0.9996**	**0.9999**	0.9997	**0.9992**	0.9978	**0.9991**	0.9987
	Single-C	Acc (%)	**99.33**	98.67	**100**	98.9	**100**	99.01	**99.33**	98	**99.33**	99.11
		MAE	**0.0041**	0.0107	**0.0038**	0.0093	**0.0019**	0.0031	**0.0089**	0.0324	**0.0017**	0.0055
	Single-R	RMSE	**0.0216**	0.0484	**0.0175**	0.0308	**0.0043**	0.0071	**0.0545**	0.092	**0.0087**	0.0225
		Corr	**0.9971**	0.9856	**0.9983**	0.9942	**0.9999**	0.9997	**0.9828**	0.945	**0.9995**	0.997

**Table 6:** Results of MPNN for each cable in case 3. The rows shaded in green indicate ten cables with tension data and values in the lower 5% of performance appear in red, and values in the upper 5% of performance appear in blue.

	MTL-Classification				MTL-Regression			Single-Classification				Single-Regression		
**Cables**	**Acc**	**Prec**	**Recall**	**F1**	**MAE**	**RMSE**	**Corr**	**Acc**	**Prec**	**Recall**	**F1**	**MAE**	**RMSE**	**Corr**
1	1	1	1	1	0.0029	0.0095	0.99953	1	1	1	1	**0.0007**	**0.0009**	0.99999
2	1	1	1	1	0.0035	0.0037	0.99998	1	1	1	1	0.0011	0.0022	0.99997
3	1	1	1	1	0.005	0.0054	0.99997	1	1	1	1	0.0009	0.0014	0.99998
4	0.97	1	0.97	0.99	0.0033	0.004	0.99996	0.97	1	0.97	0.99	0.005	0.0091	0.99944
5	1	0.97	1	0.98	0.0036	0.0038	0.99999	1	0.94	1	0.97	0.0056	0.0124	0.99955
6	1	1	1	1	0.0018	0.0023	0.99998	1	1	1	1	0.0011	0.0017	0.99998
7	1	1	1	1	0.0035	**0.0117**	**0.9993**	1	1	1	1	0.0033	0.008	0.99971
8	1	1	1	1	0.0014	0.0021	0.99997	1	1	1	1	0.0023	0.0045	0.9999
9	1	1	1	1	0.0022	0.0062	0.99977	1	1	1	1	**0.0008**	0.0014	0.99999
10	1	1	1	1	0.004	0.0103	0.99951	1	1	1	1	0.001	0.0017	0.99999
11	1	1	1	1	0.0017	0.002	0.99999	1	1	1	1	0.0021	0.0065	0.99979
12	1	1	1	1	0.0017	0.0025	0.99997	1	1	1	1	0.001	0.002	0.99998
13	**0.96**	1	**0.96**	0.98	0.0023	0.0035	0.99993	0.96	1	0.96	0.98	0.0022	0.0052	0.99989
14	**0.91**	1	**0.91**	**0.95**	0.0024	0.0031	0.99998	**0.91**	1	**0.91**	**0.95**	**0.0115**	**0.0194**	**0.9989**
15	1	**0.89**	1	**0.94**	0.0028	0.0035	0.99998	1	**0.89**	1	**0.94**	0.0065	0.0118	0.99917
16	1	1	1	1	0.002	0.0024	0.99999	1	1	1	1	0.0012	0.0022	0.99997
17	1	1	1	1	**0.0012**	**0.0014**	**1**	1	1	1	1	0.0017	0.003	0.99995
18	1	1	1	1	0.0027	0.0074	0.99972	1	1	1	1	0.0031	0.0067	0.9998
19	1	1	1	1	0.0028	0.0112	0.99943	1	1	1	1	0.0024	0.0063	0.99984
20	1	1	1	1	0.0016	0.0038	0.99992	1	1	1	1	0.0026	0.0037	0.99993
21	1	1	1	1	**0.0008**	**0.0014**	0.99999	1	1	1	1	0.0019	0.0051	0.99989
22	1	1	1	1	0.0045	0.0049	0.99993	1	1	1	1	0.0019	0.0048	0.99989
23	0.97	1	0.97	0.99	0.0054	0.0061	0.99994	0.97	1	0.97	0.99	0.0026	0.0034	0.99993
24	1	**0.95**	1	0.98	0.0014	0.0018	0.99998	1	1	1	1	0.0015	0.0025	0.99997
25	1	0.97	1	0.98	0.0028	0.0043	0.99991	0.97	0.97	0.97	0.97	0.0037	0.0069	0.99977
26	1	1	1	1	0.003	0.0046	0.99992	1	1	1	1	0.003	0.006	0.99979
27	1	1	1	1	0.0017	0.0024	0.99996	1	1	1	1	0.0015	0.0036	0.99992
28	1	1	1	1	0.0039	0.0043	0.99999	1	1	1	1	0.0031	0.0062	0.99981
29	1	1	1	1	0.0024	0.003	0.99997	1	1	1	1	0.0021	0.0044	0.99992
30	1	1	1	1	0.0018	0.0027	0.99996	1	1	1	1	0.0018	0.0038	0.99993
31	1	0.97	1	0.99	0.0049	0.0081	0.99981	1	1	1	1	0.0022	0.0044	0.99993
32	0.97	1	0.97	0.98	0.0025	0.0055	0.99988	0.97	1	0.97	0.98	0.0034	0.0068	0.99985
33	1	1	1	1	**0.0065**	0.0072	0.99995	1	0.97	1	0.98	0.0043	0.0077	0.99974
34	1	1	1	1	0.0031	0.004	0.99992	1	1	1	1	0.0047	0.006	0.99995
35	1	1	1	1	**0.0223**	**0.027**	**0.9958**	1	0.97	1	0.99	**0.0081**	**0.0127**	**0.9991**
36	1	1	1	1	0.0027	0.003	0.99998	1	1	1	1	0.0019	0.004	0.99992
37	1	1	1	1	0.0019	0.0024	0.99997	1	1	1	1	0.0013	0.0017	0.99999
38	0.97	1	0.97	0.98	0.0044	0.0053	0.99994	0.97	1	0.97	0.98	0.001	0.0016	0.99998
39	1	1	1	1	0.0023	0.0026	0.99997	1	1	1	1	0.0015	0.0024	0.99997
40	1	1	1	1	0.0029	0.0032	0.99994	1	1	1	1	0.0026	0.0035	0.99989
Q5	0.97	0.97	0.97	0.98	0.00139	0.00178	0.99942	0.96	0.95	0.96	0.97	0.0009	0.0014	0.99917
Q95	1	1	1	1	0.00545	0.01122	0.99999	1	1	1	1	0.00658	0.01241	0.99999
